# 18F-FDG PET/CT in a Case of Urothelial Carcinoma in the Urachus Presenting as Colon Cancer

**DOI:** 10.3390/diagnostics12010031

**Published:** 2021-12-24

**Authors:** Jeannette D. Andersen, Knud Fabrin, Astrid Petersen, Helle D. Zacho

**Affiliations:** 1 Department of Nuclear Medicine, Aalborg University Hospital, 9000 Aalborg, Denmark; h.zacho@rn.dk; 2 Department of Clinical Medicine, Clinical Cancer Research Center, Aalborg University, 9000 Aalborg, Denmark; 3 Department of Urology, Aalborg University Hospital, 9000 Aalborg, Denmark; knf@rn.dk; 4 Department of Pathology, Aalborg University Hospital, 9000 Aalborg, Denmark; acp@rn.dk

**Keywords:** urachal cancer, 18F-FDG PET/CT, urothelial carcinoma

## Abstract

Urachal cancer arises from an embryologic remnant of the urogenital sinus and allantois and accounts for approximately 1% of bladder malignancies. The most encountered histologic subtype is adenocarcinoma. We present a 76-year-old man suspected to have an advanced sigmoid cancer infiltrating nearby organs. A supplemental 18F-FDG PET/CT showed high tracer uptake in a tumorous process coherent with the dome of the bladder wall involving the sigmoid colon. Cystoscopy revealed a normal bladder wall, except for a small edematous area in the anterior bladder. Biopsies from the sigmoid colon and transurethral resection from the bladder confirmed a urothelial carcinoma originating from the urachus.

**Figure 1:**
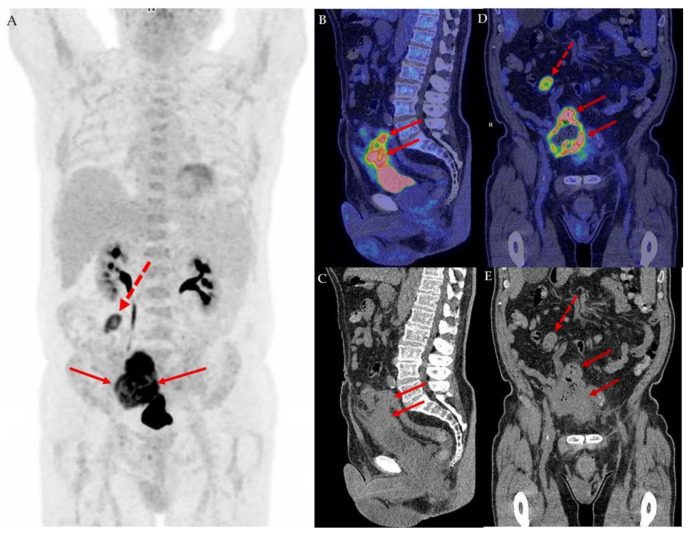
We present a 76-year-old man initially suspected to have an advanced colon cancer infiltrating nearby organs including the bladder and small intestines. During colonoscopy, the operator describes an ulcerating tumor in the sigmoid colon occupying more than half of the circumference. Surprisingly, the biopsies revealed a urothelial carcinoma. A supplemental 18F-FDG PET/CT conducted as part of the Danish standard-of-care for staging of muscle invasive bladder cancer was conducted along with a cystoscopy [1,2]. The maximum intensity projection of the 18F-FDG PET/CT image in the anterior view (**A**) revealed a high tracer uptake in an infiltrating tumorous mass coherent with the top of the bladder (full arrows) and an enlarged metastatic lymph node with high tracer uptake (dotted red arrow) in the upper right abdomen. In the sagittal view of the fused 18F-FDG PET/CT image (**B**) and low-dose CT (**C**), the coherence of the tumor to the top of the bladder wall stretching along the urachus is elegantly illustrated, supporting the suspicion of a urachal cancer. The tumor infiltrates the peritoneum, small intestines and sigmoid colon. The intestinal involvement is apparent in the coronal fused 18F-FDG PET/CT image (**D**) and coronal low dose CT (**E**), which shows the tumor (full arrows) and lymph node metastasis (dotted arrow). The cystoscopy revealed no visible tumors and a normal bladder wall. However, a small area in the top of the anterior bladder wall looked edematous. A small transurethral resection of 3 ml tissue from this area fulfilled the criteria for the diagnosis of urothelial carcinoma, high-grade, originating from the urachus, except that no urachus remnants were found in the specimen [3]. The stage according to the Sheldon Classification [4] was IVA and according to the TNM 8th Edition [5] and TNM Supplement 5th Edition [6] pT4a.

**Figure 2:**
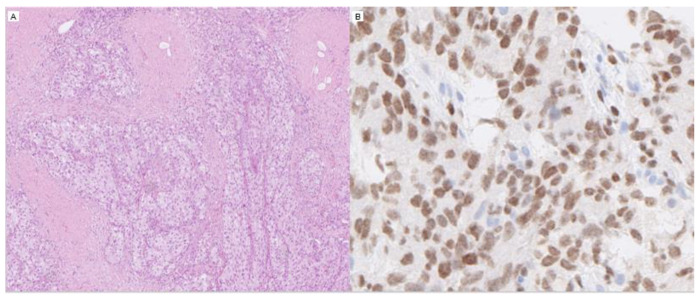
Two histopathological images of urothelial carcinoma, H&E, low magnification (**A**), and GATA3, high magnification (**B**).

Urachal cancer is a rare entity which accounts for 0.5–2% of bladder cancer [7] and most urachal malignancies represents an adenocarcinoma [7,8,9,10,11,12]. According to Reis et al, 58 cases have been reported during the last [6,7] decades, representing a urothelial carcinoma [13]. We present a case of an unusual presentation of the disease which illustrates how an 18F-FDG PET/CT elegantly complements the diagnosis and staging.

Due to the infiltrating tumorous growth involving the sigmoid colon, small intestines and lymph node metastases no primary tumor resection was performed. The patient was offered palliating oncologic treatment.

## Data Availability

Not applicable.
